# Generative models of living development: an interaction-centered proposal and its implications for artificial life research

**DOI:** 10.3389/fsysb.2026.1904510

**Published:** 2026-09-25

**Authors:** Bin Yin, Xiao-Rui Wu

**Affiliations:** 1 School of Psychology, Fujian Normal University, Fuzhou, China; 2 School of Education, Fuzhou University of International Studies and Trade, Fuzhou, China

**Keywords:** active inference, artificial life, code biology, developmental systems, human-rat interaction paradigm, layered agency, normativity, teleonomy

## Abstract

Artificial life and integrative systems neuroscience increasingly seek to explain how agency is organized through development, not merely how an already-formed controller optimizes a predefined task. This article advances an interaction-centered theory that links computational generative models, which infer hidden causes and candidate action policies, with experimental ecologies, which generate the developmental histories to be explained. The framework distinguishes reward and prior preference from concern, normativity, habit, and action policy. Concerns denote system-relative dimensions of regulatory significance grounded in an agent’s organization; habits are historically acquired dispositions; and action policies remain formally defined candidates within a computational model. The code-mediated concern hypothesis proposes that events acquire functional significance when adaptor-implemented mappings make cues informative about viability-linked concerns, thereby changing inference, precision, habit, and action selection. Teleonomic and organizational accounts locate the normative force of these concerns in present self-maintaining organization rather than conscious design or backward causation. The Layered Model of Agency differentiates operative, reflective, joint, material-mediated, sign-mediated, and symbolic agency, preventing evidence for low-level control from being generalized to collective, artifact-mediated, symbolic, or phenomenal agency. The Human-Rat Interaction Paradigm (HRIP) supplies an extensible experimental architecture for studying how structured human-rat interaction histories shape multidomain developmental trajectories. The inaugural male-rat study permits only sex-specific inference; the broader HRIP paradigm accommodates female, male, and mixed-sex samples in comparative and mechanistic research designs. Six propositions and eight predictions define tests of model comparison, cue remapping and adaptor perturbation, habit-policy dissociation, reciprocal coupling, material and sign mediation, multimodal trajectories, developmental and sex moderation, and layer-specific evidence. Together, these elements define a testable program for developmental artificial life while preserving the distinction between functional agency-like organization and phenomenal agency.

## 1 Introduction

Artificial life combines an engineering ambition with a theoretical one: constructing systems whose organization clarifies what life and agency require. Cellular automata, evolutionary simulations, embodied robots, world models, and neural agents have shown that complex adaptive behavior can arise from simple rules, recurrent coupling, and self-organization (Langton, 1989; Beer, 1995; Bedau, 2003; Ha and Schmidhuber, 2018). Embodied and enactive approaches further established morphology, sensorimotor coupling, and organism-environment organization as explanatory resources rather than implementation details (Varela et al., 1991; Clark, 1996; Pfeifer and Bongard, 2006; Froese and Ziemke, 2009). Many artificial systems nevertheless enter evaluation as already-formed controllers facing externally specified tasks. Their competence may be substantial, yet the developmental organization through which events acquire significance remains outside the model.

Developmental agency is the formation, stabilization, differentiation, and reorganization of situated capacities for action across a history of organism-environment interaction. Task competence concerns whether a system can achieve a specified outcome. Developmental agency additionally concerns how the system comes to register differences as opportunities, threats, obligations, invitations, or resources; how prior interactions sediment into habits; how its organization establishes criteria of better and worse; and how individual, joint, and mediated forms of action become possible. Developmental systems theory and probabilistic epigenesis offer a compatible foundation because they explain phenotype and behavior through reciprocal relations among genetic, neural, behavioral, social, and environmental processes rather than through a privileged causal level (Gottlieb, 2007).

Integrative systems neuroscience provides an empirical framework for this target by connecting molecular and cellular processes, neural circuits, bodily regulation, behavior, social interaction, and environmental feedback. Its emphasis on multiscale modeling, emergent phenomena, multimodal measurement, and systems-level explanation converges with the synthetic methods of artificial life (Chang, 2024). This convergence is most informative when the situations that generate data preserve developmentally relevant relations. A brain-behavior correlation collected in a brief task and an artificial agent trained against a reward function may each omit the history that makes the observed action intelligible.

Generative models enter this program in two complementary senses. In the computational sense, a generative model specifies how hidden states and causes give rise to observations and how beliefs and action policies are inferred. In the experimental sense, a developmental ecology generates the histories and observations from which causal and computational models are estimated. This second sense concerns the organization of data generation rather than data volume alone. Partner identity, reciprocity, contingency, predictability, bodily state, affordances, artifacts, signs, and the temporal ordering of interaction determine which developmental processes can occur and which can later be identified.

Treating the experimental arrangement as part of the explanation raises the question of how developmental meaning is constituted. Neither temporal extension nor physical stimulation alone determines what an event signifies for a living system. System-relative significance arises when an implemented mapping makes an event informative about conditions that matter to the system’s organization. Code biology supplies one candidate mechanism: adaptors implement correspondences between distinguishable domains, so a mapping is neither a retrospective observer label nor a direct physicochemical necessity (Barbieri, 2015; 2018). Vedor (2026) extends this logic to psychological meaning-making and relates codepoiesis, the production and conservation of such mappings, to free-energy formulations. The code-mediated concern hypothesis treats these mappings as testable mechanisms without presuming that all developmental meaning is code-like or that such adaptors have already been demonstrated.

Clarifying meaning also requires a disciplined vocabulary of action. In reinforcement learning and active inference, policy denotes a candidate sequence or conditional course of action. Extending the term to habits, norms, joint commitments, tool-mediated capacities, and symbolic practices obscures important differences in organization and invites confusion with governance or public policy. Action policy is therefore reserved for the formal computational object. Habit, norm-guided action, action organization, and disposition describe broader biological and cognitive phenomena. Keeping these categories distinct makes it possible to relate low-level action selection to situated meaning, joint action, abstract thought, and artifact-mediated agency without reducing them to a single mechanism.

These distinctions are organized by the Layered Model of Agency developed by Mendoza-Collazos and Zlatev (2022) and elaborated for collective agency by Mendoza-Collazos (2025a), Mendoza-Collazos (2025b). The model differentiates operative, reflective, joint, material-mediated, sign-mediated, and symbolic agency. Higher layers depend on and reorganize lower capacities instead of adding complexity to a common policy-selection mechanism. The model also places subjectivity at an evidential boundary: within its cognitive-semiotic formulation, genuine agency entails at least minimal subjectivity. The conventional engineering term artificial agent remains useful for systems that sense, learn, and act, but it does not settle whether those systems possess genuine or phenomenal agency.

The Human-Rat Interaction Paradigm (HRIP) provides an empirical platform on which parts of this theory can be progressively tested (Wu et al., 2025). As a paradigm, HRIP organizes developmentally timed human-rat interaction histories and relates them to later emotional, cognitive, social, physiological, and neural trajectories. Its inaugural implementation assigned one-month-old male Sprague-Dawley rats to positive, negative, or ordinary early-adolescent conditions. Designated human caretakers delivered these conditions over 3 weeks, after which the rats underwent multidomain behavioral assessment. The resulting evidence is specific to that sampled population, whereas the paradigm leaves sex, developmental period, interaction structure, and measurement modality as open design factors. The initial study illustrates the developmental logic, while subsequent implementations can provide more discriminating tests of reciprocity, partner specificity, meaning remapping, and layered agency.

Sections 2–5 define the core constructs, distinguish the layers of agency, connect them to efficient, formal, and teleonomic explanation, and situate HRIP within developmental rodent research. Sections 6, 7 derive six propositions, eight discriminating predictions, and an experimental blueprint for artificial life and integrative systems neuroscience.

## 2 Core vocabulary and the interaction-centered hypothesis

### 2.1 From task success to system-grounded normativity

A task defines success relative to an instruction, benchmark, or designer objective. Such criteria are indispensable in engineering, but they do not by themselves establish system-grounded normativity. Living systems continually encounter conditions under which their organization is sustained, damaged, repaired, or transformed. Energetic sufficiency, bodily integrity, thermoregulation, controllability, social access, and recovery participate in the conditions that allow the system to remain capable of action. Autopoietic and enactive accounts connect these conditions to self-maintenance, adaptivity, sense-making, and agency (Maturana and Varela, 1980; Di Paolo, 2005; Thompson, 2007; Di Paolo et al., 2017). Barandiaran et al. (2009) accordingly identify normativity, together with individuality and interactional asymmetry, as a defining condition of minimal agency: an autonomous organization regulates its environmental coupling according to norms that sustain its continued organization.

Within this framework, normativity is an organized capacity to distinguish better from worse states, transitions, or actions according to standards that are not exhausted by an observer’s score. At basal biological levels, these standards concern viability and regulation. At social and cultural levels, they can include jointly sustained expectations, conventions, obligations, and symbolic criteria, some of which conflict with immediate biological advantage. A person may accept hunger to honor a promise or scientific norm, and a collective may reorganize an institution around values that do not reside in any single body. The Layered Model of Agency distinguishes basal, interpersonal, and symbolic forms of normativity while specifying how they can interact.

The distinction between reward and concern follows from this account. In reinforcement learning, reward is a formally defined signal used to update action values or optimize return; it may be scalar or vector-valued, sparse or dense, extrinsic or intrinsic. The term alone does not indicate whether the signal is generated by, or integrated with, the agent’s maintenance dynamics. Active inference already offers a richer formalization through prior preferences, uncertainty, and expected free energy (Friston, 2010; Parr et al., 2022; Friston et al., 2023; Da Costa et al., 2024; Pezzulo et al., 2024). In common discrete-state formulations, a C vector represents prior preferences over outcomes, and hierarchical preferences can formalize multidimensional, learnable, and context-dependent homeostatic or allostatic regulation (Pezzulo et al., 2015; Tschantz et al., 2022). Concern addresses the constitutive and developmental question of what gives these quantities normative force; it does not replace them.

A concern variable denotes a system-relative dimension of regulatory significance together with the implemented relations that connect observations and actions to it. The construct directs attention to how an organized vulnerability gives a preference normative force, how cues become evidence about that vulnerability, how those mappings are conserved or revised during development, and how stable reorganization can be distinguished from transient response or externally imposed valuation. In active-inference models, concern-related effects may be distributed across prior preferences, likelihood and transition models, habitual priors, precision, and hierarchical state representations. Concern thus provides a constitutive and developmental interpretation of a coordinated set of formal quantities rather than a new scalar objective.

Evidence for concern must scale with the strength of the attribution. In biological organisms, the relation between regulation and viability provides an initial basis. In artificial systems, merely assigning a variable a label such as energy, pain, or social need is insufficient. A stronger functional attribution requires the variable to participate in maintaining operative organization, constrain action across multiple domains, create counterfactual vulnerability when perturbed, and remain modifiable through the system’s own history. These criteria establish functional concern only. Whether a system experiences concern remains a separate question.

### 2.2 Action policy, habit, and norm-guided action

Within this vocabulary, policy retains a restricted technical meaning. An action policy, denoted π , is a candidate sequence or conditional organization of actions evaluated under a model. Reinforcement learning typically defines a policy as a mapping from states or observations to action probabilities, whereas active inference evaluates candidate policies in relation to expected free energy and prior beliefs about policies. This technical definition should not be extended to every stable behavioral tendency.

A habit is a history-dependent disposition that makes some actions or action sequences more available, probable, fluent, or resistant to reconsideration. Active-inference models can represent habits as learned priors over policies generated through repeated action and can examine how habitual and goal-directed control interact (Friston et al., 2016). In a broader biological sense, habits include sedimented sensorimotor routines, affective expectations, postural tendencies, and relational styles. They can bias formal policy selection without being identical to a selected policy, and they may organize action before an explicit choice set is represented. A history of reliable support may sediment as flexible approach and rapid recovery, whereas unpredictable threat may sediment as vigilance, hesitation, or rigid avoidance across contexts. Describing such trajectories only as newly selected policies would obscure their embodied and historical organization.

Norm-guided action is the broader category. It includes action organized by viability conditions, learned expectations, shared commitments, conventions, or symbolic standards. Operative habits, reflective plans, joint commitments, tool-mediated practices, and linguistic reasons can all guide action normatively, but their architectures differ. Computational models may represent aspects of each through policies and priors without implying that every layer literally consists of policies. Table 1 summarizes these distinctions.

**TABLE 1:** Terminological disambiguation for the proposed theory.

Term	Operational definition	Role in the theory	Boundary or misuse to avoid
Reward	A formally specified signal used to update action values or optimize return	Engineering objective or learning signal; can be scalar or vector-valued	Does not by itself establish that the objective is generated by, or integrated with, the system’s self-maintenance
Prior preference/C vector	A distribution over preferred outcomes or states in discrete active-inference formulations	Represents expected desirability and can implement multidimensional, context-dependent homeostatic or allostatic targets	Should not be described as merely scalar reward; its normative grounding is not fixed by the parameter alone
Action policy ( π )	A candidate sequence or conditional organization of actions evaluated under a formal model	Precise computational object for reinforcement learning or active inference	Not a synonym for every stable behavior, disposition, social norm, or public policy
Habit (h)	A history-dependent disposition that increases the availability, probability, fluency, or persistence of actions	Links developmental history to later action and can bias attention, precision, and action-policy selection	Not identical to a single selected policy; may organize action before explicit deliberation
Concern variable (c)	A latent system-relative dimension of regulatory significance plus the implemented relations that make observations and actions relevant to it	Interprets distributed preference, precision, transition, habit, and regulatory variables in relation to an organized vulnerability	Not a new scalar reward; not warranted merely because a variable is labeled pain, energy, or social need
Normativity	The system-grounded distinction between better and worse states, transitions, or actions relative to operative or jointly sustained standards	Connects viability, skilled success, social expectations, conventions, and explicit norms across agency layers	Not reducible to observer scoring; higher social and symbolic norms need not maximize immediate homeostasis
Code/adaptor	A physically implemented correspondence between distinguishable domains, mediated by an adaptor so that alternative mappings are possible	Candidate mechanism by which cues acquire concern-relevant significance and can be selectively remapped	Conventionality does not imply conscious agreement; a code claim requires an identifiable and manipulable mediator
Meaning/significance	An event’s system-relative relevance when an implemented mapping or interaction process makes it evidence about concerns and reorganizes action	Links developmental ecology to belief, precision, habit, and norm-guided action	Functional significance does not by itself establish phenomenal experience, and sense-making need not always be code-like

### 2.3 How significance is constituted: codes, adaptors, and codepoiesis

Code biology gives a specific account of how a cue can become relevant to a concern. An organic code consists of correspondences between entities in two independent domains, implemented by a third class of entities called adaptors (Barbieri, 2015; Barbieri, 2018). In this technical context, conventional means that the correspondence is not fixed by direct physical necessity and that alternative mappings are physically possible; it does not imply conscious agreement. The genetic code is the paradigmatic case because adaptor molecules implement correspondences between nucleotide triplets and amino acids. Whether comparable codes operate at neural or psychological levels remains an empirical question.

Vedor (2026) extends code biology to psychological phenomena and relates codepoiesis, the continuing production and conservation of codes, to free-energy formulations. The code-mediated concern hypothesis specifies how such mappings affect inference: a developmental event acquires system-relative functional significance when a maintained mediator maps a cue or relational pattern onto consequences for one or more concerns, thereby altering evidence, precision, habitual disposition, or action-policy selection. The resulting account locates functional significance in the relation among cue, mapping, and concern rather than in the cue alone and yields a direct empirical requirement: changing the mapping should change the cue’s functional significance while leaving basic sensory discrimination intact.

A mechanistic code claim therefore requires an identifiable and manipulable adaptor. Correlation alone is insufficient. The candidate system must contain at least two distinguishable domains, a mapping not reducible to direct physical necessity, and a mediator whose alteration changes that mapping while preserving relevant sensory and motor capacities. Receptor configurations, neuromodulatory gates, circuit assemblies, and learned sensorimotor routines are candidate mediators rather than established organic codes. Artificial implementations might use routing modules, learned binding systems, context gates, or memory-mediated translators. If no distinct mediator can be identified, or if associative and dynamical models predict the same interventions without a code construct, the code claim provides no additional mechanistic leverage.

The approach of a human hand illustrates the problem. Retinal motion and air displacement contribute efficient causes, but they do not determine whether the event signifies warmth, capture, play, restraint, food, or threat. Repeated relations among approach cues, partner identity, controllability, bodily change, and outcome can establish differentiated mappings. A cue may then become high-precision evidence of danger after one history and weak or positive evidence of connection after another. Computationally, the change may be expressed in likelihood mappings, transition expectations, prior preferences, policy priors, or precision. Developmentally, it changes what the cue affords and how the organism is prepared to act.

Functional significance must remain distinct from phenomenal meaning. In the cognitive-semiotic account, genuine agency entails subjectivity (Mendoza-Collazos and Zlatev, 2022), so a functional mapping cannot establish what, if anything, the system experiences. Code-mediated mappings also need not exhaust meaning. Dynamical coupling, affective attunement, and participatory sense-making may generate significance through processes that do not decompose into discrete sign-to-meaning correspondences (De Jaegher and Di Paolo, 2007). Codes are treated here as one experimentally tractable mechanism within a wider ecology of sense-making.

### 2.4 Generative models as computational objects and experimental ecologies

The two senses of generative model are methodologically coupled but not identical. A computational generative model is a probabilistic or dynamical object that estimates hidden causes and predicts the sensory consequences of action. A generative experimental ecology is a causal arrangement that produces developmental histories and observations. Their connection lies in identifiability: the ecology determines which latent changes can occur and be distinguished, while the computational model expresses hypotheses about those changes. Brief, decontextualized tasks often leave developmental explanations observationally equivalent. The same avoidance response may reflect acute fear, a cue-specific learned value, generalized threat mapping, a rigid habit, social distrust, impaired motor capacity, or contextually appropriate caution. History, transfer, perturbation, and recovery are needed to separate these possibilities.

Causal texture denotes the trajectory-preserving dependency structure among the elements of a developmental ecology: cues, partner identity, timing, contingency, predictability, bodily and neural state, candidate adaptor state, concern-related variables, habits, action policies, environmental consequences, recovery, and cross-context transfer. No single dataset must measure every level. The concept instead guides design by making explicit which dependencies are represented and which omissions restrict inference. Longitudinal and multimodal measurements deepen the causal account only when they are synchronized with interaction events and individual trajectories.

Generative developmental ecologies also change the interpretation of variability. Between-individual differences are often treated as noise around a group mean, although stable divergence may be the central developmental phenomenon. Different histories can yield distinct organizations of vigilance, exploration, memory, social openness, and recovery even when mean task performance is similar. The relevant modeling target is therefore the distribution of developmental profiles, their latent organization, and their response to perturbation. In artificial life, this requires comparing the forms of flexibility, rigidity, cooperation, repair, and meaning remapping produced by different ecologies instead of ranking agents by a single terminal score.

## 3 Layered agency: from operative habits to symbolic and collective action

### 3.1 The six layers and their relations

The Layered Model of Agency was developed within cognitive semiotics to avoid both indiscriminate attribution of agency to every causally effective entity and its restriction to deliberate human choice (Mendoza-Collazos and Zlatev, 2022). It distinguishes operative, reflective, joint, material-mediated, sign-mediated, and symbolic agency. The first three constitute original agency, in which action does not constitutively depend on artifacts; the latter three constitute enhanced agency, in which material and semiotic resources expand the capacity to act.^1^ Fundierung denotes an asymmetric relation in which higher layers depend on and reorganize lower capacities. The layers do not form a scale of moral worth, and a single situated activity may recruit several at once.

Operative agency comprises embodied, often pre-reflective action in which skill and intention are expressed in the performance itself. Running, orienting, grasping, withdrawing, exploring, and regulating posture typically depend on sensorimotor organization and affective readiness without requiring explicit deliberation. Habits are central at this layer. They are not mechanical reflexes; they can be context-sensitive and modifiable, but their normativity is expressed through embodied success, failure, fluency, interruption, and recovery.

Reflective agency introduces explicit anticipation, evaluation, or commitment. The agent can compare possible actions, imagine outcomes, revise an intention, or provide a reason. Formal action-policy selection is most naturally associated with parts of this layer, although computational models can also describe operative action. The critical addition is not a longer planning horizon alone but reflexive access to alternatives and to the action as one’s own.

Joint agency arises when agents coordinate in relation to a shared or mutually regulated activity. It ranges from spontaneous interpersonal coordination to explicit shared intentions and commitments. Joint action research has shown that coordination depends on representing and predicting one’s own and others’ actions, while enactive work emphasizes that the interaction process can acquire partial autonomy and shape individual sense-making (Sebanz et al., 2006; De Jaegher and Di Paolo, 2007; De Jaegher et al., 2010). A joint action cannot always be reconstructed by adding two independent policies. Reciprocity, role differentiation, repair, and shared normativity can become properties of the coupled system.

Material-mediated agency occurs when artifacts become constitutive resources for action. A tool, interface, notation surface, or built environment does not merely add an external stimulus; it reorganizes the agent’s possible operations and can stabilize memory, attention, coordination, and control. In the cognitive-semiotic account, the artifact is not thereby an independent genuine agent. Its agency is derived through incorporation into the activity of subjective agents. For artificial-life research, this layer motivates experiments in which agents learn to appropriate, redesign, and jointly maintain artifacts rather than merely select fixed tools.

Sign-mediated agency recruits nonlinguistic signs and sign systems to guide action across absent, counterfactual, or socially shared situations. Gestures, diagrams, maps, icons, and conventional signals can reorganize attention and coordination. Their meaning cannot be inferred from token use alone; it is demonstrated through systematic interpretation, transfer, repair of misunderstanding, and productive recombination. Creative meaning events occur when an agent or group establishes a novel but intelligible mapping, not simply when it reproduces a trained association.

Symbolic agency recruits linguistic and other symbolic systems that support abstraction, explicit norms, narratives, institutions, and reasons. Symbolic agency can distance action from immediate affordances and biological needs, allowing agents to formulate principles, negotiate obligations, and coordinate across long temporal and social scales. Mendoza-Collazos (2025a), Mendoza-Collazos (2025b) uses the layered model to analyze collective agency as enhanced action within sociotechnical systems, including how individual values contribute to shared norms. Table 2 translates these layers into empirical requirements and artificial-life design targets.

**TABLE 2:** Empirical requirements and artificial-life design targets for the Layered Model of Agency. Adapted from the six-layer agency hierarchy of Mendoza-Collazos and Zlatev (2022), with collective-agency elaborations from Mendoza-Collazos (2025a), Mendoza-Collazos (2025b); modified and extended for empirical and artificial-life testing.

Layer	Defining capacity	Normativity/meaning	Empirical or artificial-life test	Evidence from the inaugural HRIP study
1. Operative	Embodied skill, affective readiness, habits, regulation, and action without constitutive reliance on artifacts	Viability, fluency, interruption, skilled success/failure, and recovery	Sensorimotor regulation; habit revision; transfer across contexts; perturbation and recovery	Directly assessed: History-dependent operative dispositions and multidomain transfer
2. Reflective	Explicit anticipation, comparison of alternatives, plans, reasons, and self-attribution	Standards can be represented, compared, justified, and revised	Counterfactual planning; sensitivity to reasons; ownership judgments; metacognitive report where available	Not directly assessed; rodent work offers limited access to explicit alternatives or self-attribution
3. Joint	Mutually responsive coordination organized around shared or jointly regulated activity	Shared expectations, roles, commitments, error correction, and repair	Reciprocal prediction; contingent-versus-yoked design; role change; coordination recovery; partner specificity	Not directly assessed; partner-specific behavior is relevant, but stronger evidence requires reciprocal designs
4. Material/artifact-mediated	Tools and built resources become constitutive of action and expand the reachable action space	Functional norms become stabilized in appropriated and maintained artifacts	Appropriation, flexible reuse, redesign, and performance changes when the artifact is removed or transformed	Not assessed; future HRIP designs could test appropriation and flexible artifact use
5. Sign-mediated	Nonlinguistic signs, maps, diagrams, gestures, and conventions guide action across absent or shared situations	Publicly interpretable mappings, transfer, and repair of misunderstanding	Novel sign negotiation; systematic transfer; productive recombination; repair after ambiguity or convention change	Not assessed; future designs would require evidence of convention, transfer, and repair
6. Symbolic	Language and symbolic systems support abstraction, explicit norms, institutions, narratives, and reasons	Norms can be formulated, contested, justified, and transformed	Compositional generalization; counterfactual reasoning; explicit norm representation; reason-giving	Beyond the plausible scope of rodent HRIP and not inferable from task performance

### 3.2 Normativity and meaning change across layers

Treating all six layers through one undifferentiated value function would obscure how normativity changes across them. At the operative layer, better and worse are expressed primarily through embodied regulation, skilled coping, and viability. At the reflective layer, standards can be represented and compared. At the joint layer, success depends on mutually responsive coordination and shared expectations. At material-mediated and sign-mediated layers, norms become stabilized in artifacts, conventions, and publicly inspectable structures. At the symbolic layer, agents can formulate, contest, and transform the norms themselves.

Concerns and habits also change their organization across layers. Basal concerns constrain action through bodily vulnerability. Social concerns develop through histories of dependence, affiliation, recognition, and exclusion. Symbolic concerns can be constituted through identities, institutions, and ideals whose force cannot be reduced to immediate homeostasis. Habits likewise sediment at every layer, including bodily routines, styles of reflection, turn-taking patterns, tool practices, interpretive conventions, and discursive forms. Developmental theory must therefore explain how a layered repertoire of habits and norms is acquired, coordinated, and revised, not simply which action policy is selected.

The code-mediated concern hypothesis is correspondingly layer-specific. At operative layers, adaptor mappings may connect sensory and interoceptive patterns to viability-related significance. At joint and mediated layers, mappings can be socially negotiated, materially stabilized, and publicly corrected. At symbolic layers, codes are explicitly conventional and can themselves become objects of reflection. Biological codes, learned cue mappings, cultural conventions, and formal symbol systems should not be conflated because they differ in origin, mechanism, and validation criteria. Their shared feature is implemented correspondence, demonstrated through evidence appropriate to the layer.

### 3.3 Engineering agents, genuine agency, and the subjectivity boundary

Artificial-life research commonly uses the term agent for robots, software processes, and simulated organisms that sense, act, learn, and alter an environment. The cognitive-semiotic model uses agency more restrictively because it treats agency and subjectivity as complementary aspects of intentionality (Mendoza-Collazos and Zlatev, 2022). Behavioral performance alone cannot establish genuine agency. These two usages serve different purposes and should remain distinct.

Three levels of attribution help preserve this distinction. Agentic functionality denotes autonomous coordination of sensing and action relative to internal variables or goals. Agency-like organization adds developmental integration with self-maintenance, normativity, historical individuality, and reciprocal coupling. Phenomenal or genuine agency requires adequate evidence of a first-person perspective or sense of agency. Synthetic systems can demonstrate the first two. The third remains an open empirical and philosophical question, especially for artificial systems.

This distinction supports a substantive artificial-life program. A system that preserves itself, acquires concern-sensitive mappings, forms habits, coordinates with others, appropriates artifacts, and uses signs productively would constitute a substantial theoretical result even if subjectivity remained unresolved. Such a system could reveal organizational conditions necessary for richer forms of agency and serve as a synthetic model for biological comparison. Fluent behavior, ungrounded linguistic self-report, or anthropomorphic description would remain insufficient grounds for inferring experience (Lin, 2025; Shanahan, 2024).

### 3.4 Methodological implications: perspective-matched evidence

Mendoza-Collazos and Zlatev (2022) frame agency research as a conceptual-empirical loop supported by pheno-methodological triangulation. Theoretical distinctions guide operationalization, and empirical findings in turn revise those distinctions. In human research, first-person reports, second-person interaction, and third-person behavioral and neural measures should constrain one another. A neuroscience paradigm based on isolated button presses or manipulated action-outcome timing may be internally reliable while offering limited construct validity for joint, material-mediated, sign-mediated, or symbolic agency. A neural correlate of action attribution is therefore evidence about a component of agency, not the location of agency as a whole.

Second-person neuroscience and participatory paradigms study mutually responsive interaction instead of passive observation (Schilbach et al., 2013). The same principle extends beyond human neuroscience. Nonhuman research cannot rely on first-person reports, but it can combine fine-grained ethology, partner-specific behavior, microtemporal reciprocity, physiological regulation, neural dynamics, and counterfactual perturbation without claiming direct access to subjective experience. In artificial systems, complete access to internal variables remains third-person mechanistic evidence rather than a substitute for first-person perspective. Evidential standards must therefore match both the agency layer and the claim.

## 4 Efficient, formal, and teleonomic causality

### 4.1 Three explanatory questions

Developmental agency raises three complementary causal questions. Efficient causes identify mechanisms and events that produce change, such as sensory input altering neural activity, prediction error updating a model, hormonal modulation of circuit excitability, or parameter change altering behavior. Formal causes identify the organization within which those mechanisms operate, including bodily morphology, network architecture, interaction topology, environmental layout, temporal pattern, and developmental ecology. Teleonomic finality identifies the system-relative ends or norms according to which a change counts as regulation, failure, repair, or development. None of these questions substitutes for the others.

The same efficient event can have different consequences under different formal organization. Handling can be supportive, neutral, or threatening depending on timing, controllability, partner identity, and prior history. The same behavioral output can have different teleonomic roles. Freezing may protect against immediate threat, express a rigid generalized habit, or interrupt exploration that would otherwise improve long-term viability. Explanation requires the mechanism, the organized context, and the norm relative to which the action is evaluated.

Formal causality is especially important for generative experimental ecologies. The unit of manipulation is not always a single stimulus. It can be a repeated relation: whether a partner’s approach is contingent on the subject’s behavior, whether interruption is predictable, whether a tool remains available across contexts, or whether social repair follows conflict. These higher-order structures constrain which habits, meanings, and forms of agency can emerge. An experiment that randomizes individual events while destroying their relational organization can eliminate the phenomenon it intends to measure.

### 4.2 Teleonomy and naturalized organizational teleology

Intentional or external teleology explains purposiveness by appeal to conscious intention, deliberate design, or an intended end. Naturalized or organizational teleology instead explains purposiveness through the current organization of a living system, without invoking conscious intention or backward causation. Teleonomy refers to pre-subjective or non-conscious goal-oriented organization in self-maintaining, regulatory, and adaptive systems. Pittendrigh (1958) introduced the term to describe the apparent purposiveness of adaptations without treating future outcomes as efficient causes. Mayr (1974) distinguished teleonomic programs and goal-directed processes from cosmic teleology, and contemporary work has reopened questions about purpose, agency, and innovation in living systems (Corning et al., 2023). Within this tradition, directionality can be studied scientifically while the organization of purposiveness within a living system remains an empirical and theoretical question.

Organizational accounts address that question. Bich (2024) explicitly calls this an organizational teleology: a naturalized account that grounds biological purposiveness in regulatory control within self-maintaining organization. The term does not denote conscious intention or an externally imposed end. On this view, a process is purposive not merely because it was historically selected, but because it currently contributes to maintaining the organization that produces and regulates it. Di Paolo’s (2005) notion of adaptivity similarly links teleology to the capacity to distinguish and regulate tendencies that move the system toward or away from viability. Evolutionary history, current organization, and ontogenetic learning therefore operate at different but connected timescales.

Teleonomic finality can integrate these timescales without positing an additional force. Phylogeny and historical construction establish families of viable organization and available mappings. Organizational closure gives some variables current normative force because their disruption threatens continued operation. Ontogeny calibrates habits and mappings through interaction. Moment-to-moment inference and action then unfold under these constraints. Teleonomic organization becomes empirically tractable through counterfactuals: deviation from a viable range recruits regulation, repeated failure of a relational mapping promotes its revision, and violation of a shared norm elicits repair or sanction. Efficient mechanisms realize these responses, formal organization constrains them, and teleonomic analysis explains why they count as regulation or repair.

### 4.3 From teleonomic organization to inference

The relations among observation, context, concern, mapping, and history can be stated schematically. Let **o_t_** denote observations, **x_t_** relational and environmental context, **c_t_** a vector of concern-relevant conditions, A_t_ a candidate adaptor or mapping state, and H_t_ the accumulated interaction history. Table 3 lists possible operationalizations and the evidential status of each variable. System-relative functional significance is represented as a mapping from observation and context relative to concern organization and developmental history:

$$\mu_{t}=\Phi_{A_{t}}\left(o_{t},x_{t},c_{t};H_{t}\right).$$ (1)

**TABLE 3:** Formal variables, operational interpretations, and epistemic status. Boldface denotes vector-valued quantities.

Symbol	Interpretation	Possible measurement or implementation	Epistemic status/boundary
$o_{t}$	Observation vector at time t	Sensory, behavioral, physiological, neural, social, or artificial-agent observations	Standard modeling variable; modality-specific operationalization required
$x_{t}$	Relational and environmental context at time t	Partner identity, contingency, affordances, artifact/sign state, and task or ecology metadata	Theory-specific aggregation; components are directly measurable
$c_{t}$	Concern-relevant condition vector	Latent or measured viability-linked variables, including energetic, damage, uncertainty, recovery, and social-reliability dimensions	Proposed constitutive construct; not equivalent to a scalar reward or to felt concern
$A_{t}$	Candidate adaptor or mapping state	A manipulable mediator that implements a non-necessary cue/context-to-significance correspondence and indexes the observation likelihood, for example, $P\;\left(o_{\tau }\;|\;s_{\tau },\;A_{t}\right),\;A_{t}$	Strong code claim requires selective intervention, remapping evidence, and predictive gain over a model without an adaptor-indexed likelihood
$\mu_{t}$	System-relative functional significance	A latent quantity inferred from cross-domain effects on precision weighting, belief updating, transfer, recovery, and action organization	Theory-specific explanatory target; Equation 2 makes its precision link explicit, but it does not establish phenomenal meaning
$H_{t}$	Accumulated developmental interaction history	Ordered records of partners, timing, contingency, outcomes, perturbations, repair, and prior internal states	Directly recordable in principle; representation is model-dependent
π	A candidate action policy	A formally specified action sequence or conditional course of action	Standard computational object; not a synonym for habit, norm, or public policy
$E_{t}\left(\pi \right)$	Prior probability of policy π (habit term)	Learned policy-prior parameter estimated from repeated action or fitted behavior	Standard active-inference quantity; interpretation depends on model specification
$G_{t}\left(\pi \right)$	Expected free energy of policy π conditional on adaptor-indexed evidence	Model-based estimate of anticipated risk/utility and epistemic value under policy π, with the observation likelihood indexed by At	The baseline quantity is standard active inference; the explicit Gt (π; At) dependency is a theory-specific, testable extension
$\gamma_{t}$	Significance-sensitive expected-free-energy precision, γt (μt)	Confidence weighting on expected free energy as a specified function of system-relative functional significance	Precision is standard in active inference; dependence on μt is theory-specific and must earn predictive support
$e_{t}\left(\pi \right)$	Illustrative policy-count or concentration parameter	A Dirichlet-style count used in one possible implementation of habit learning	Illustrative implementation only; not required by the theory
$h_{t}$	Broader habitual state or disposition vector	History-dependent sensorimotor, affective, attentional, and relational dispositions estimated across contexts	Theory-specific bridge construct; must be distinguished empirically from current policy evaluation

Equation 1 treats $\mu_{t}$ as dependent on mapping state, context, concern organization, and history rather than on stimulus energy alone. Equation 2 closes the formal link: functional significance modulates expected-free-energy precision, while adaptor state indexes the observation likelihood. In a common discrete active-inference formulation, the initial distribution over candidate policies can be written schematically as:

$$Q_{0,t}\left(\pi \right)=\sigma \left[\ln E_{t}\left(\pi \right)‐\gamma_{t}\left(\mu_{t}\right)G_{t}\left(\pi ;A_{t}\right)\right].$$ (2)

In Equation 2, $E_{t}\left(\pi \right)$ is a learned prior over policy π that can encode habit, $G_{t}\left(\pi \right)$ is expected free energy, $\gamma_{t}$ is expected-free-energy precision, and σ denotes softmax normalization (Friston et al., 2016; Smith et al., 2022; Parr et al., 2022). The baseline policy-selection form is standard active inference. The explicit dependencies *γ_t_ (μt) and G_t_ (π; A_t_*) are theory-specific commitments: the first makes precision significance-sensitive, while the second indexes the observation likelihood by adaptor state, for example, *P (oτ | sτ, A_t_*). These dependencies can be rejected if they do not improve prediction of selective remapping or perturbation effects beyond simpler accounts.

Habits can remain analytically distinct from selected policies. One illustrative implementation accumulates a policy-specific concentration or count parameter and normalizes it to form a prior:

$$e_{t+1}\left(\pi \right)=e_{t}\left(\pi \right)+\eta_{h}\;1\left[\pi_{t}=\pi \right].$$ (3)

$$E_{t+1}\left(\pi \right)=\frac{e_{t+1}\left(\pi \right)}{\sum_{\pi^{\prime }}e_{t+1}\left(\pi^{\prime }\right)}.$$ (4)

Equations 3,4, [Disp-formula e4] illustrate one possible implementation rather than a requirement of the theory. Other learning rules may prove more appropriate. The developmental claim is that consequences can update both a candidate mapping and a broader habitual state as a function of history:

$$A_{t+1}=U_{A}\left(A_{t},o_{t},x_{t},\Delta c_{t+1};H_{t}\right).$$ (5)

$$h_{t+1}=U_{h}\left(h_{t},\pi_{t},\Delta c_{t+1};H_{t}\right).$$ (6)

In Equations 5,6, [Disp-formula e6] $U_{A}$ and $U_{h}$ are unspecified update operators, and $\Delta c_{t+1}$ denotes the concern-relevant consequence observed at the next step. The notation specifies a family of rival models; it does not assume that biological organization, subjective experience, and computation share a single formal description. A standard active-inference model may explain the data through preferences, likelihoods, transitions, policy priors, and precision without a distinct adaptor. A code-mediated model adds explanatory value only when it predicts selective cue remapping, partner-specific transfer, or targeted perturbation effects beyond simpler associative, dynamical, or active-inference accounts.

Teleonomic organization enters inference through these relations rather than through an additional causal force. The organization of $c_{t}$ determines which deviations and outcomes are normatively relevant; $A_{t}$ indexes which observations count as evidence about those deviations and how they enter the likelihood; functional significance modulates expected-free-energy precision; $h_{t}$ carries history-dependent dispositions; and policy evaluation represents prospective action under the current model. Efficient mechanisms realize the relations, formal organization constrains them, and teleonomic analysis explains why particular transitions constitute regulation, damage, repair, or success for the organized system. Figure 1 integrates developmental history, significance mapping, inference, action, and the three causal registers.

**FIGURE 1:**
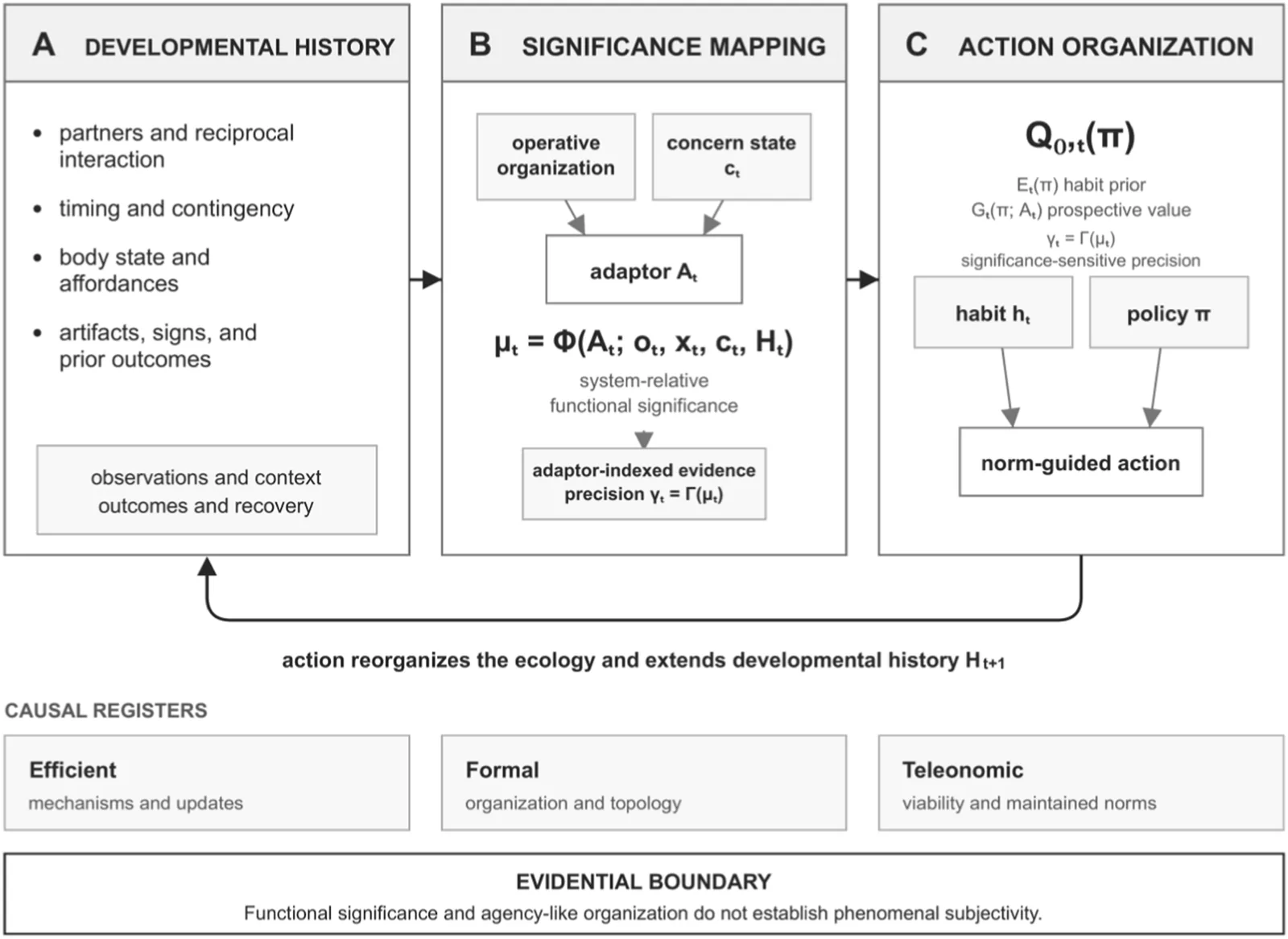
Formal architecture of interaction-centered developmental agency. Developmental history includes partners, reciprocity, timing, bodily conditions, affordances, artifacts, and signs. A candidate adaptor maps observations and context onto system-relative functional significance in relation to concern organization and accumulated history. Functional significance modulates expected-free-energy precision, while adaptor state indexes the observation likelihood and expected-free-energy term. Habit and prospective action policy remain analytically distinct influences on norm-guided action, which in turn reconstructs the ecology and extends developmental history. The lower bands distinguish efficient, formal, and teleonomic causal registers. The evidential boundary indicates that functional significance and agency-like organization do not, by themselves, establish phenomenal subjectivity. Equations 1–6, introduced in Section 4.3, and Table 3 define the formal notation and epistemic status of each variable.

## 5 HRIP as an extensible developmental paradigm

### 5.1 Paradigm architecture and inaugural implementation

HRIP defines a family of experiments in which developmentally timed human-rat interactions are systematically organized and related to later behavioral and physiological trajectories (Wu et al., 2025). The paradigm is characterized by structured interaction histories, designated human partners, longitudinal intervention, and multidomain assessment. Sex, strain, age, interaction valence, contingency, partner structure, and outcome modality remain open design dimensions. HRIP is therefore a research architecture rather than a fixed protocol, and its scope is determined by the causal contrasts that its implementations can support.

The inaugural study enrolled 48 one-month-old male Sprague-Dawley rats and assigned 16 animals to each of three conditions: positive early-adolescent experience, negative early-adolescent experience, or ordinary laboratory control. Designated human caretakers delivered the three-week intervention, and interaction behavior stabilized by the second week. Later assessments sampled anxiety and exploration, adaptation to novelty, learning and memory, responses to toy and unfamiliar rats, behavior in trapped-peer and food-access tasks, and social competition. Because the published report included no *a priori* power calculation, group contrasts should be treated as preliminary estimates rather than precise effect-size benchmarks. The male-only sample further limits inference to males; generalization across sexes requires replication in adequately powered, sex-inclusive designs with planned sex-by-history tests. HRIP itself remains open to female, male, and mixed-sex implementations and to comparisons across developmental periods.

The inaugural design is theoretically informative because it manipulates an organized history and examines distributed consequences. Because valence, predictability, controllability, bodily handling, environmental stimulation, partner identity, and relational consistency vary together, however, the composite conditions cannot identify the independent contribution of each component. Subsequent HRIP studies should therefore use factorial decomposition, trajectory modeling, transfer tests, and mechanistic follow-up. Designated partners make partner-specific generalization, cue remapping, recovery, and persistence of acquired dispositions experimentally accessible. Figure 2 distinguishes the general paradigm, the evidential scope of the inaugural study, and the priority causal tests.

**FIGURE 2:**
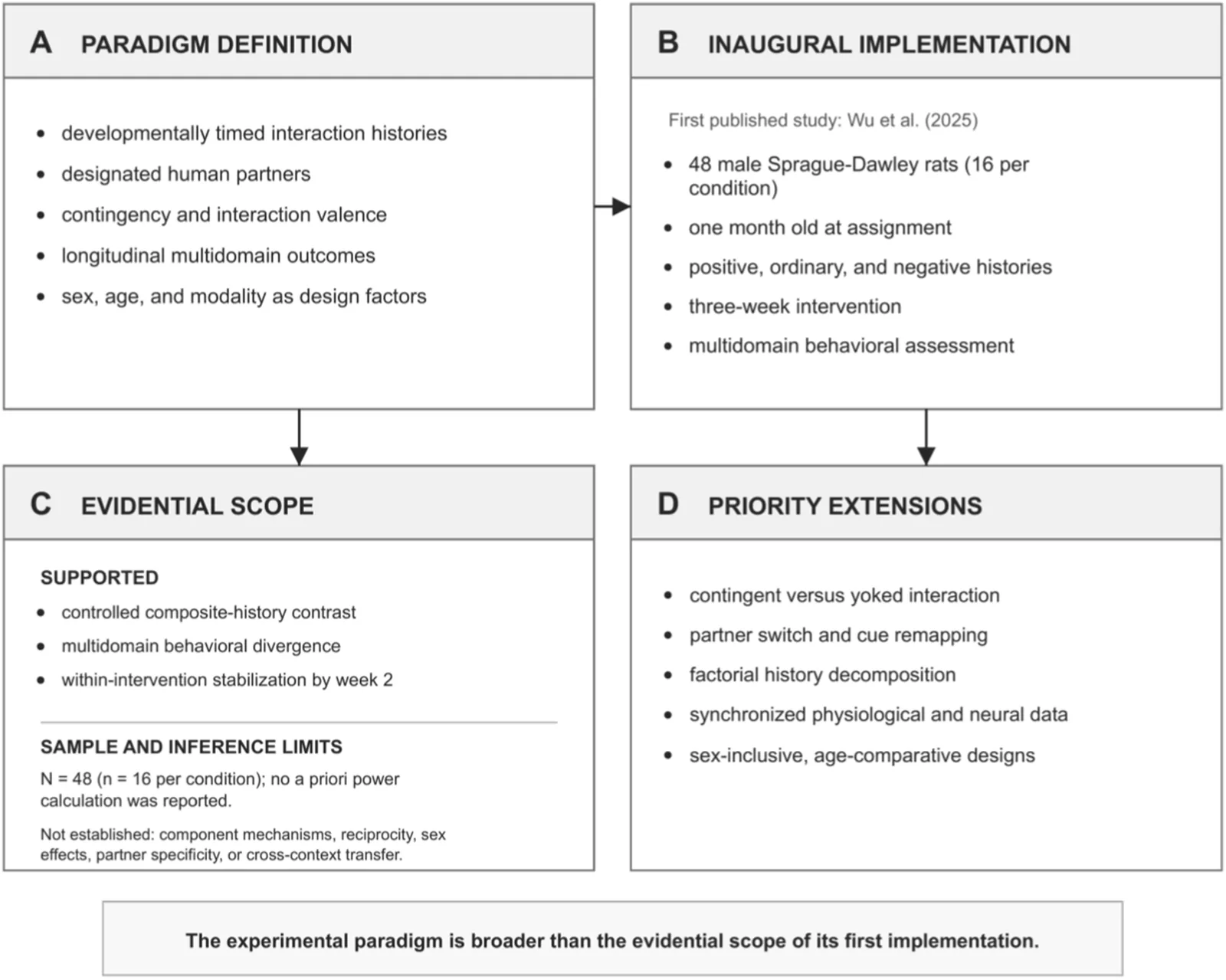
HRIP as an extensible experimental paradigm. **(A)** defines the paradigm’s general architecture: developmentally timed, structured human-rat interactions with designated partners, longitudinal intervention, and multidomain outcomes. **(B)** summarizes the inaugural study, which used 48 one-month-old male Sprague-Dawley rats, 16 per condition, and three early-adolescent histories over 3 weeks (Wu et al., 2025). **(C)** states that no *a priori* power calculation was reported and distinguishes the evidence provided by that modest, sex-limited sample from questions it leaves open. **(D)** identifies extensions that can test contingency, partner specificity, mechanism, developmental timing, and sex-related moderation. The evidential limits in **(C)** apply to the inaugural study, whereas **(A)** defines the broader design space of HRIP.

### 5.2 Relation to early-life stress and environmental enrichment

The inaugural HRIP study becomes more interpretable when situated within established rodent models. Maternal separation, limited bedding, and related early-life stress procedures manipulate disruption, scarcity, or adversity during sensitive periods and have documented enduring, sex-dependent changes in neural and behavioral development (Speranza et al., 2024; Honeycutt et al., 2020). Environmental enrichment increases physical, cognitive, and social stimulation relative to standard housing and can alter behavior and neurochemistry, although protocols vary considerably (Simpson and Kelly, 2011). These literature jointly support the broader proposition that developmental timing, caregiving conditions, stimulation, and stress shape later phenotype.

Against this background, HRIP adds a relationally organized early-adolescent intervention that includes positive, negative, and ordinary histories and assigns designated human caretakers. This architecture enables questions about security, connection, partner specificity, and generalization that are difficult to pose with deprivation or diffuse enrichment alone. Operational overlap remains substantial: positive HRIP conditions include enrichment-like features, and negative conditions include stress-like features. Direct comparison is therefore needed before observed differences can be attributed specifically to relational organization, meaning, or concern. Stronger designs would cross general stimulation with relational contingency, match stress load while varying predictability, and compare designated-caretaker interaction with equivalent nonrelational object exposure.

Developmental systems theory treats intervention effects as products of reciprocal organism-environment relations rather than as unidirectional environmental inputs. Outcomes arise through reciprocal relations among the rat’s prior organization, its ongoing behavior, the caretaker’s responses, cage-level social dynamics, and later test contexts. A probabilistic-epigenetic account therefore predicts heterogeneous trajectories and cross-level interactions rather than uniform programming (Gottlieb, 2007). The central question is which configurations of organism and ecology produce stable yet revisable forms of regulation.

### 5.3 Controlled histories and reciprocal coupling

The ecological ambition of HRIP does not require abandoning experimental control. HRIP remains a controlled environmental intervention assessed primarily through third-person behavior, and it is compatible with behavior-analytic description. Its distinctive contribution lies in treating the organized interaction history, rather than an isolated stimulus, as the unit of developmental manipulation.

When organized history becomes the unit of analysis, the rat’s ongoing behavior can shape each encounter, designated partners make relational specificity testable, and outcomes can be assessed through multidomain trajectories, transfer, and recovery rather than immediate response strength alone. These features allow direct tests of constructivist and ecological hypotheses. A stronger interaction-centered implementation would quantify reciprocal microdynamics, model the rat as an active source of variation, and manipulate coupling itself, for example, by comparing contingent and yoked caretaker responses while holding nominal valence constant.

Contingent-yoked and partner-switch designs make the ecological claim empirically discriminating. In a yoked comparison, one rat receives caretaker events contingent on its behavior, while another receives the same events and timing without contingency. A stimulation account predicts similar outcomes; an interaction-centered account predicts divergence because controllability and reciprocity alter the relation among action, partner, and consequence. A partner-switch test can similarly distinguish generalized arousal from relational learning. Such manipulations preserve experimental control while testing whether coupling structure has causal force.

### 5.4 Agency layers accessible to HRIP

Within the Layered Model, evidence from the inaugural HRIP study bears most directly on operative agency, including embodied exploration, avoidance, feeding, vigilance, learning, and recovery. The paradigm can be extended to rudimentary joint organization by measuring reciprocal coordination with caretakers or conspecifics. The published study did not establish mutually regulated shared action, reflective agency, material appropriation, conventional sign use, or symbolic agency. Mapping HRIP onto the layered model therefore identifies both the processes already accessible and the design changes required to investigate additional layers.

Inferences must remain matched to the construct actually tested. A task can measure one component of agency reliably while offering limited validity for agency as a whole (Mendoza-Collazos and Zlatev, 2022). Behavioral profiles from the inaugural study provide evidence about developmental organization, not direct measures of subjective meaning or sense of agency. For nonhuman animals, stronger triangulation can combine partner-specific approach, contingency sensitivity, coordination repair, physiological co-regulation, neural and endocrine dynamics, and selective transfer. Convergence across these measures would support stronger but still layer-specific claims about agency.

### 5.5 Generalizing beyond the inaugural implementation

Generalization across sex is an empirical question because early-life and adolescent experience can produce sex-dependent behavioral, neural, and connectivity effects in rodents (Honeycutt et al., 2020; Speranza et al., 2024). Future HRIP studies can address this question through adequately powered sex-inclusive designs, planned sex-by-history tests, explicit modeling of developmental timing, and measurement-invariance analysis for latent trajectories. Cycle-related variation should be measured or modeled when it is relevant to the outcome and design rather than used as a reason to exclude females.

Other dimensions of generality include strain, species, developmental period, laboratory, caretaker characteristics, and social housing arrangement. Each extension probes a different boundary condition. Human surrogate caregiving is not homologous to human child development, so translational claims require convergent evidence from other animal paradigms, human longitudinal and interactive studies, and synthetic models with explicit mechanisms. HRIP contributes a manipulable comparative framework rather than a direct replica of human development.

## 6 The interaction-centered developmental agency theory

### 6.1 Six linked propositions

The distinctions developed in Sections 2–4 yield six linked but separately testable propositions concerning concern, habit, action policy, layered agency, and the three causal registers.

Proposition 1: Organizational normativity. Developmental agency requires distinctions between better and worse states or actions that are grounded in the system’s operative organization or, at higher layers, in jointly maintained and symbolic norms. An externally assigned score is not sufficient. Evidence requires showing that perturbing the relevant variable changes maintenance, regulation, or norm-guided action across contexts.

Proposition 2: Code-mediated significance. Some developmental meanings arise through manipulable adaptor systems that map cues or relational patterns onto concern-relevant consequences. The mapping is not fixed by the cue’s physical properties and can be selectively remapped. Support requires evidence for a distinct mediator and a physically non-necessary correspondence; in their absence, associative or dynamical explanations remain sufficient.

Proposition 3: Historical sedimentation. Repeated interaction changes not only momentary action policies but also habits: embodied and inferential dispositions that bias future perception, action, and recovery. Habits are expected to transfer across tasks when they concern a common relational or regulatory dimension, but to remain revisable under sustained counterevidence.

Proposition 4: Ecological generation. The formal organization of a developmental ecology, including reciprocity, contingency, predictability, partner identity, affordances, artifacts, signs, and temporal order, constrains which meanings and agency layers can emerge. Equal event frequencies do not imply equivalent development when relational structure differs.

Proposition 5: Layered emergence. Operative, reflective, joint, material-mediated, sign-mediated, and symbolic agency require partially distinct organizations and evidence. Higher layers are founded on and transform lower layers. Successful low-level control neither demonstrates nor fully explains joint or symbolic agency.

Proposition 6: Perspective-matched evidence. Claims about agency must be matched to evidence. Third-person behavior and mechanism can establish agentic functionality and organization; reciprocal interaction can support claims about joint coordination; and first-person or otherwise compelling evidence is required for phenomenal agency. No single scalar measure is valid across all layers.

Figure 1 depicts the developmental cycle specified by Propositions 1 through 4, and Figure 3 shows how Propositions 5 and 6 constrain extension to higher layers. Table 4 translates the propositions into discriminating tests.

**FIGURE 3:**
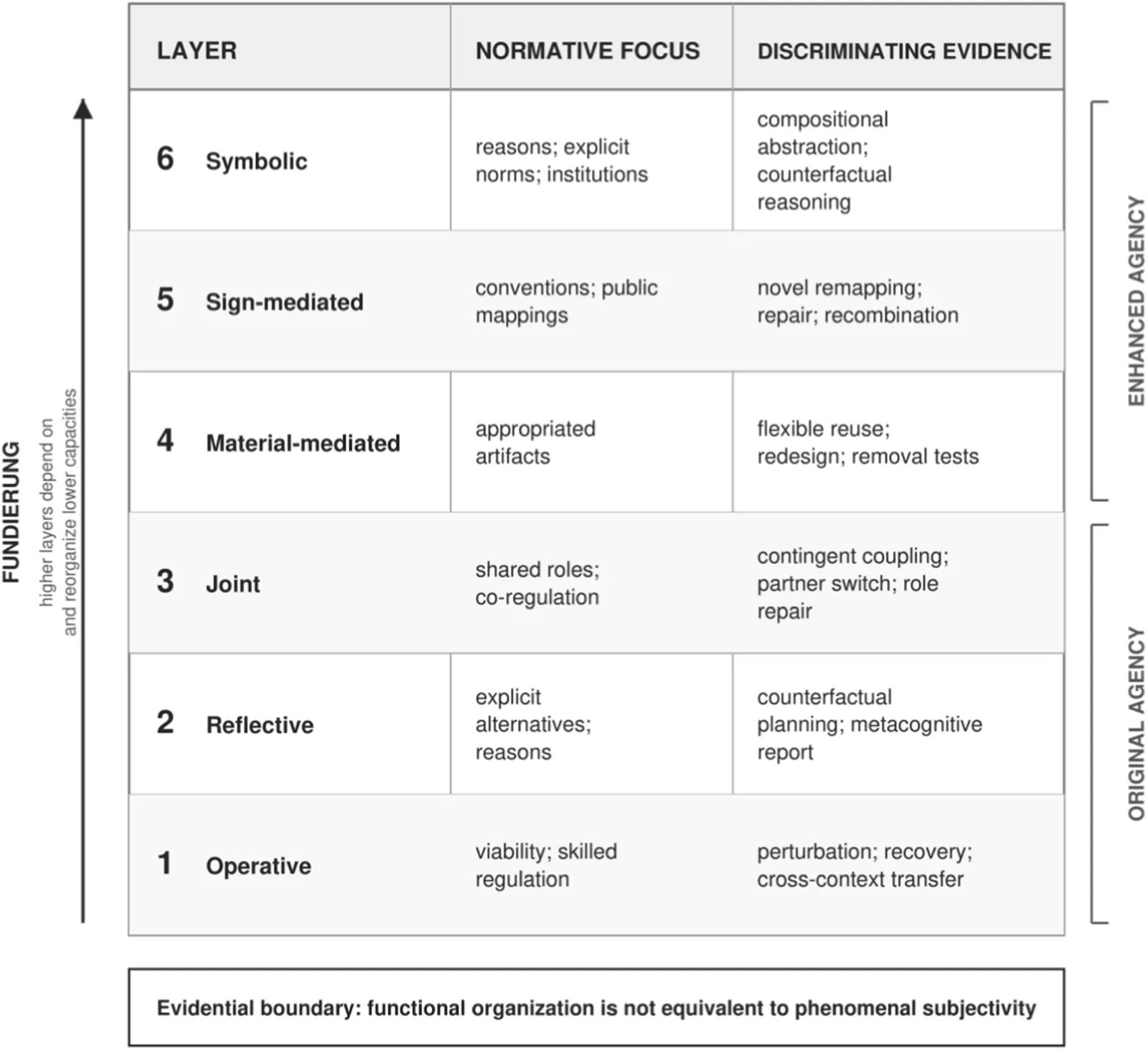
Layered agency and perspective-matched evidence. Adapted from the six-layer agency hierarchy of Mendoza-Collazos and Zlatev (2022), with collective-agency elaborations from Mendoza-Collazos (2025a), Mendoza-Collazos (2025b); redrawn and extended to specify evidential requirements and boundaries. The Layered Model distinguishes operative, reflective, joint, material-mediated, sign-mediated, and symbolic agency. Higher layers are founded on and reorganize lower capacities (Fundierung); the arrangement is not a scalar increase in agency. Layers 1 to 3 constitute original agency, in which action does not depend constitutively on artifacts, and layers 4 to 6 constitute enhanced agency, in which material and semiotic resources expand the capacity to act. Each layer introduces a distinct normative focus and requires discriminating evidence, summarized in Table 2. The lower boundary indicates that functional organization is not equivalent to phenomenal subjectivity.

**TABLE 4:** Discriminating empirical program for the interaction-centered developmental agency theory.

Prediction	Decisive design	Supportive result	Rival result or falsifier
1. Nested model comparison	Fit reward-only, standard active-inference, habit-augmented, and code-adaptor models to the same longitudinal intervention and transfer data	The code-adaptor model improves out-of-sample prediction specifically under cue remapping, partner change, or cross-context transfer, with identifiable parameters	Standard active inference with preferences, transitions, and precision predicts the interventions equally well; the code construct lacks incremental necessity
2. Cue remapping and adaptor perturbation	Reverse or recombine cue–partner–outcome relations; selectively perturb, freeze, lesion, or swap the candidate mapping mediator	Meaning follows adaptor/context state while sensory discrimination and basic motor capacity remain intact	Only global impairment occurs, or no selective mediator can be identified despite adequate tests
3. Habit–policy dissociation	Change immediate incentives, volatility, or explicit models after a developmental history; test rapid transfer to tasks sharing a latent concern	History-dependent dispositions persist or bias attention/precision independently of online action-policy evaluation, yet remain revisable under sustained counterevidence	Responses track only current contingencies or cue-specific memories with no cross-context disposition
4. Reciprocity and joint organization	Compare contingent interaction with a yoked condition matched for event frequency, intensity, and timing distribution; include partner switch and repair perturbations	Contingent histories produce partner-specific reciprocal prediction, role differentiation, co-regulation, and recovery not reducible to exposure totals	Matched exposure fully explains outcomes; coupling adds no emergent organization
5. Material and sign mediation	Compare resources present, absent, transformed, and available for redesign; require novel mapping, transfer, and repair rather than trained token response	Artifacts reorganize action possibilities; signs are negotiated, recombined, transferred, and repaired under novelty	Performance depends on memorized sequences or lookup mappings and collapses under systematic novelty
6. Multimodal latent trajectories	Collect synchronized behavioral, physiological, neural, social, and contextual time series; fit state-space, mixture, dynamical, and multilevel generative models	Latent trajectories predict transfer and intervention response beyond isolated scores and preserve individualized developmental transitions	A single task score predicts equally well, or latent profiles merely redescribe the variables used to construct them
7. Developmental timing and sex	Use adequately powered, sex-inclusive and age-stratified implementations; estimate sex-by-history and age-by-history effects and test measurement invariance	History effects or latent organizations vary by sex, developmental order, plasticity window, embodiment, or resource constraints	Developmental order and embodiment repeatedly add no explanatory value once event totals are controlled, weakening the developmental-ecology claim
8. Layer-specific evidence	Use multiple benchmarks and interventions indexed to operative, reflective, joint, material-, sign-, and symbolic agency	Profiles show partial independence and asymmetric dependence; higher layers can be disrupted selectively while basal regulation remains intact	All layers collapse to one scalar performance measure or alleged higher-layer capacities persist without the proposed founding organization

### 6.2 Prediction 1: nested model comparison

The first prediction is tested by fitting four nested model classes to the same longitudinal data: a reward-based model, a standard active-inference model with learnable preferences and transitions, a habit-augmented model, and a code-adaptor model. The code-adaptor model is supported only if it improves out-of-sample prediction specifically under cue remapping, partner change, or cross-context transfer. Better fit to training data is insufficient. Bayesian model comparison, predictive accuracy, and parameter-recovery analysis can establish whether the additional mapping state is identifiable.

If standard active inference with preferences, transitions, and precision predicts intervention and transfer effects as well as the code model, the code construct adds no demonstrable explanatory value. In that case, concern may still guide constitutive interpretation, but the mechanistic code claim should be weakened.

### 6.3 Prediction 2: cue remapping and adaptor perturbation

A decisive test reverses or recombines the relation among cue, partner, and outcome after a developmental history has been established. A code-mediated account predicts selective remapping: altering the adaptor or contextual gate changes the cue’s significance while preserving basic cue detection and motor capacity. In animals, candidate mechanisms can be perturbed pharmacologically, neurally, or through contingency retraining. In artificial systems, a routing or binding module can be lesioned, swapped, or frozen directly.

Support would require preserved sensory discrimination alongside loss or remapping of the cue’s concern-related effect according to adaptor state rather than physical similarity. A global performance loss would not support the claim. Failure to identify a selective mediator after theory-guided perturbation of plausible candidates would count against the code hypothesis.

### 6.4 Prediction 3: habit-policy dissociation

Evidence for a habit-policy dissociation arises when a developmental history leaves a disposition that differs from online action-policy evaluation. Such a disposition persists under changed immediate incentives, appears rapidly across tasks sharing a latent concern, or biases attention and precision before explicit choice. Goal-directed or reflective action remains more sensitive to current contingencies and explicit model changes. Manipulations of volatility, retraining, or contextual novelty can test whether the two forms of control dissociate.

In HRIP research, the relevant prediction is not simply that a negative history increases avoidance. A threat-shaped history should produce a cross-context disposition, such as elevated vigilance, delayed recovery, or altered partner generalization, that cannot be reduced to memory for a single punished action. In artificial systems, identical current likelihood, transition, and preference models can be combined with different learned habit priors to test whether history alters exploration and recovery independently of present reward.

### 6.5 Prediction 4: reciprocity and emergent joint organization

Interaction-centered theory predicts that reciprocal coupling produces outcomes not explained by matched event exposure. Contingent and yoked conditions should therefore diverge even when the frequency, intensity, and timing distribution of individual events are controlled. Evidence for joint organization includes reciprocal prediction, role differentiation, error correction, co-regulation, and recovery of coordination after perturbation.

For artificial life, two agents trained independently and later paired should be compared with agents whose histories require co-development. The latter should display partner-specific coordination and repair that cannot be reconstructed by summing independent policies. At higher levels, shared norms should be inferred only when agents maintain a coordination rule across role changes, sanction or repair violations, and can communicate or renegotiate the rule.

### 6.6 Prediction 5: material and sign mediation expand, rather than merely trigger, agency

A material or sign resource constitutes part of agency when its acquisition reorganizes the reachable action space, memory, coordination, or abstraction. Mere response to a trained token is not sufficient. Tests compare performance with the resource present, absent, transformed, and available for redesign. Material-mediated agency predicts appropriation and flexible use of artifacts; sign-mediated agency predicts interpretation, transfer, repair of misunderstanding, and productive recombination.

Novel mapping provides a stricter test: an agent or group should establish a mapping under shared constraints and reuse it in a new context. A lookup table or memorized sequence can mimic success on familiar trials but should fail under systematic novelty. Symbolic agency requires stronger evidence: compositional generalization, counterfactual reasoning, explicit norm representation, or reason-giving cannot be inferred from fluent output alone.

### 6.7 Prediction 6: multimodal latent trajectories

If concerns and habits organize multiple domains, longitudinal multimodal data should reveal latent trajectories that predict behavior better than isolated scores. Relevant animal channels include movement kinematics, posture, freezing, ultrasonic vocalization, social distance, feeding, heart rate, endocrine measures, and neural activity. Artificial analogues include action sequences, uncertainty estimates, energy budgets, communication, memory states, environmental modification, and recovery dynamics.

The theory predicts structured covariance and individualized transitions rather than a single valence factor. A security-linked trajectory might combine broad exploration with rapid physiological recovery and flexible partner generalization. A threat-linked trajectory might combine vigilance, selective memory, feeding interruption, and slow recovery. Mixture models, state-space models, dynamical systems identification, and multilevel generative models should be compared. Latent profiles should predict transfer and intervention response, not merely summarize the measures used to construct them.

### 6.8 Prediction 7: developmental timing and sex moderate organization

Developmental ecologies may produce different organizations across periods and sexes. In HRIP and related biological paradigms, adequately powered designs can estimate sex-by-history and age-by-history interactions and test measurement invariance of latent variables. A profile estimated in one sex requires validation before generalization to another. Artificial systems offer a synthetic analogue: altering developmental order, plasticity windows, embodiment, or resource constraints may produce different stable organizations despite equal total exposure. Because path dependence is central to living development, consistent failure of developmental order, embodiment, or sex to add explanatory value after event totals are controlled would count against the theory’s emphasis on developmental ecology.

### 6.9 Prediction 8: layer-specific evidence prevents construct collapse

Experimental outcomes should dissociate across layers. An agent may show robust operative control without reflection, joint coordination without symbolic reasoning, or fluent symbol use without grounded concern-sensitive regulation. The Layered Model predicts that no single benchmark will rank these capacities validly. Multi-benchmark profiles should therefore display partial independence and asymmetric dependence: disruption of operative capacities should impair higher layers, whereas removing a sign system may leave basal regulation intact while impairing abstract or collective action.

For neuroscience, experiments should map specific constructs and perspectives rather than search for a unitary neural locus of agency. For artificial life, layer labels should be earned through interventions appropriate to each layer. Passing a symbolic task supports competence under that test; it does not establish subjectivity. Results in which all proposed layers collapse to one scalar performance, or in which higher-layer behavior persists without the founding organization specified by the model, would count against the layered account.

## 7 A blueprint for artificial life and integrative systems neuroscience

### 7.1 Designing developmental artificial-life ecologies

An interaction-centered artificial-life program specifies an ecology alongside an objective function. The design identifies what the system maintains, which partners and affordances are available, how interactions are contingent, which artifacts and signs can be appropriated, and how plasticity changes over developmental time. Counterfactual histories are compared within a shared architecture, while alternative architectures encounter matched histories. This factorial logic separates organizational effects from cumulative exposure.

At the operative layer, synthetic systems need internal variables whose perturbation has distributed consequences, sensorimotor embodiment, adaptive regulation, and the capacity to form revisable habits. At the joint layer, environments should require reciprocal coordination, partner recognition, role change, and repair. At material-mediated layers, artifacts should be transformable and capable of reorganizing action rather than serving as fixed inputs. At sign-mediated and symbolic layers, systems should negotiate mappings, detect misunderstanding, compose signs, and coordinate around explicit or publicly stabilized norms. Figure 3 presents these as layer-specific design problems, not as a single scale of intelligence.

The framework complements work on enactive artificial intelligence, developmental robotics, diverse and collective intelligence, autotelic agents, and open-endedness (Froese and Ziemke, 2009; Lehman and Stanley, 2011; Cangelosi and Schlesinger, 2015; Levin, 2022; Colas et al., 2022; McMillen and Levin, 2024; Hughes et al., 2024). Autotelic goal generation expands the space of objectives, but self-generated goals acquire normative significance only when they are constrained by the system’s organization, formed through consequential interaction, integrated across domains, and revisable through meaning-making. Open-endedness likewise concerns the emergence of new norms, practices, artifacts, and signs, not behavioral novelty alone.

Contemporary generative systems can display impressive interactive competence without acquiring it through their own developmental histories. World models learn interactive simulators, large-model agents produce socially plausible behavior, and foundation-scale systems generate controllable environments (Ha and Schmidhuber, 2018; Park et al., 2023; Bruce et al., 2024). Competence inherited from human data, prompts, or design scaffolds should therefore not be treated as evidence of developmentally grounded agency. Evaluation should distinguish capacities constituted through the system’s own embodied and relational history from those that depend on human semantic infrastructure and specify what evidence would justify stronger claims.

### 7.2 Data architecture and analysis

Testing the framework requires longitudinal, synchronized, multimodal, context-sensitive data that preserve individual trajectories. Each observation should be linked to partner identity, action contingency, environmental state, relevant artifact or sign, internal variables, and subsequent recovery or transfer. Individual identities and microtemporal sequences should be retained rather than replaced by group averages, and the data architecture should record missing modalities explicitly so that causal claims remain calibrated to what was measured.

Analysis combines mechanistic intervention with generative modeling. State-space and dynamical models can identify latent trajectories; active-inference models can formalize beliefs, preferences, precision, habits, and action policies; causal models can test mediators and counterfactual histories; and code models can be compared when a candidate adaptor is specified. Greater model complexity is warranted only by out-of-sample prediction, perturbation response, parameter recovery, and acceptable identifiability.

### 7.3 A reciprocal program with systems neuroscience

Integrative systems neuroscience supplies biological constraints and experimental paradigms, whereas artificial life supplies synthetic systems in which architectures, histories, and internal variables can be manipulated with unusual precision. Animal and human data can rule out synthetic mechanisms that fail to reproduce developmental trajectories; synthetic models can test the sufficiency of proposed organizations, generate counterfactual predictions, and identify measurements that distinguish rival explanations.

The layered account also changes neuroscience design. Operative agency calls for embodied and regulatory measures; joint agency requires interactive paradigms and coordination dynamics; mediated and symbolic agency require artifact use, sign processes, and sociocultural context. In human research, pheno-methodological triangulation can integrate first-person, second-person, and third-person evidence with neural measurement. In nonhuman research, second-person-like reciprocal designs and ethological evidence can improve ecological and construct validity without implying direct access to subjective experience.

## 8 Discussion

The theory locates developmental agency in the historically organized system formed through agent-environment interaction rather than in an isolated controller. Computational models articulate latent causes and candidate actions, whereas experimental ecologies determine which histories, mappings, habits, and forms of coordination can arise and be identified. Concern and normativity specify why some differences matter to the system; code-mediated mappings provide one testable route by which cues acquire functional significance; and the Layered Model constrains inference from operative control to joint, mediated, symbolic, or phenomenal agency. Developmental ecology thus constrains what can emerge, while perspective-matched evidence constrains what the observations warrant.

Computational models and experimental ecologies are distinct kinds of generative model, but their coordination is methodologically productive. An ecology that preserves relevant dependencies improves identification of computational hypotheses, whereas a computational model clarifies which interventions and measurements distinguish rival developmental explanations. The shared terminology is justified only when it yields sharper empirical designs.

The proposed mechanisms do not exclude standard associative learning, dynamical systems, enactive sense-making, or active inference, which may explain many developmental effects without organic codes. The code-mediated concern hypothesis adds explanatory value only when selective mediators and physically non-necessary mappings can be identified. Concern variables may organize inquiry without corresponding to a single neural variable, but their distributed character does not exempt them from empirical failure. Teleonomic explanation is similarly bounded: it accounts for system-relative directionality without implying conscious intention, moral value, or inevitable progress. Norms can conflict across timescales and levels, as when an acute stress response preserves immediate safety while damaging long-term health or an institutional norm sustains a collective while harming individuals.

The Layered Model was developed primarily for cognitive-semiotic analysis of human and animal agency, so its use in artificial life remains a theoretical extension. Its strong subjectivity criterion is contested by more inclusive enactive accounts that attribute genuine agency to minimal living systems (Barandiaran et al., 2009). The distinction among agentic functionality, agency-like organization, and phenomenal agency allows synthetic research to investigate the first two without settling the third by definition.

HRIP connects the theory to experimental work without reducing the paradigm to its inaugural implementation. In that male sample, structured early-adolescent interaction histories were associated with divergent multidomain behavioral profiles. Its composite conditions, third-person measures, and distance from human development delimit what the data establish. The broader paradigm can be expanded through factorial manipulation of reciprocity, predictability, stimulation, partner specificity, and stress; synchronized physiological and neural measurement; cue-remapping and transfer tests; and sex-inclusive, developmentally comparative implementations. A cumulative program depends on preserving the distinction between paradigm architecture and implementation-specific evidence.

No single artificial-life model needs to instantiate every layer. Minimal systems can isolate organizational normativity; dyads can test emergent coordination; artifact ecologies can test enhanced action; and sign games can test conventional remapping. Each model should support only claims commensurate with its design and evidence. Applying living agency to any adaptive controller erodes the term’s precision, but uncertainty about subjectivity does not preclude investigation of functional and organizational forms of agency.

## 9 Conclusion

Generative models of living development comprise computational models that infer hidden causes and experimental ecologies that generate causally interpretable histories. Their integration requires an account of organizational normativity, code-mediated significance, habit formation, teleonomic directionality, and layered agency. Action policy remains a formal computational construct; habits and norm-guided action describe historically organized dispositions and practices. HRIP offers an extensible architecture for testing how structured human-rat interactions shape developmental trajectories, while its inaugural implementation provides initial evidence rather than a fixed template. Across biological and artificial systems, the framework directs inquiry toward how operative, joint, mediated, and symbolic forms of agency emerge, how interventions alter them, and what evidence warrants each attribution. Functional organization and phenomenal agency remain distinct explanatory targets.

## Footnotes

1 The six-layer hierarchy and the original/enhanced distinction follow the peer-reviewed account of Mendoza-Collazos and Zlatev (2022), licensed under CC BY 4.0. For collective-agency elaborations unavailable there, the discussion also draws on Mendoza-Collazos (2025a), a Spanish-language book chapter, and its English OSF preprint (Mendoza-Collazos, 2025b), which is non-peer-reviewed and licensed under CC BY 4.0.
